# The Impact of Manner Adverb on the Gestural Embodiment of Actions Described by Literal and Metaphoric Sentences

**DOI:** 10.3390/bs13020155

**Published:** 2023-02-11

**Authors:** Omid Khatin-Zadeh, Danyal Farsani, Jiehui Hu, Zahra Eskandari, Hassan Banaruee

**Affiliations:** 1 School of Foreign Languages, University of Electronic Science and Technology of China, Chengdu 610054, China; 2 Department of Teacher Education, Norwegian University of Science and Technology, 7491 Trondheim, Norway; 3 Department of English, Chabahar Maritime University, Chabahar 99717-56499, Iran; 4 Department of English, American, and Celtic Studies, University of Bonn, 53115 Bonn, Germany

**Keywords:** embodiment, gesture, literal sentence, metaphoric sentence

## Abstract

The aim of this study was to examine the impact of manner adverbs on the gestural embodiment of actions that are described by literal and metaphoric sentences. We asked a group of participants to read and then orally retell four stories. Each story had two versions. In one version, literal and metaphoric sentences describing literal and metaphorical actions did not include manner adverbs. In the other version of each story, the same sentences included a manner adverb that provided more information about literal or metaphoric actions. Participants’ reproductions of stories were recorded with a camera and were analyzed to make a comparison between gestures that accompanied sentences that included a manner adverb and sentences that did not include a manner adverb. The results showed that when literal and metaphoric sentences included a manner adverb, there was a higher probability of using a gesture than when these sentences were used without a manner adverb. In other words, using a manner adverb increases the probability of using a gesture with literal and metaphorical sentences. Therefore, it is suggested that adding a manner adverb to a literal or metaphoric sentence can strengthen the process of embodiment of the action described in that sentence. We present two explanations for this observation.

## 1. Introduction

Embodiment theories and embodied language cognition have been the subject of a continuously enlarging volume of works in recent years. Among these studies, the embodiment of verbs (actions) has been at the focus of attention of many behavioral studies. However, the embodiment of other categories of words such as manner adverbs has been less examined by behavioral investigations. Manner adverbs are one category of words that provide information about the manner of an action. For example, in the sentence *he walks slowly,* the word *slowly* provides more information about the action of the verb. In this study, we aimed to examine how these words are embodied in gestures when they are used in literal and metaphoric sentences. Embodiment is the shared area of many disciplines, including cognitive science, cognitive psychology, cognitive linguistics, education, computer sciences, and philosophy. According to the key assumption of embodied language cognition, when people use language to talk about an experience, the same sensorimotor processes involved in perceiving that experience are reactivated; e.g., [1,2,3,4,5]. This means that actually perceiving an experience and using language to talk about that experience involve the same neural networks and the same sensorimotor processes; e.g., [4,6,7,8]; for reviews, see [9,10]. For example, if an experience involves seeing an object, visual networks and visual processes involved in seeing the object are activated when the individual talks about that experience. Interestingly, some evidence has suggested that even when the individual imagines an object, without using language to talk about it, the visual processes associated with seeing the object are activated. In one of these studies, Farah [11] found that visual imagery and the real visual perception of an object involve the activation of the same areas in the visual system in the prestriate occipital cortex, parietal, and temporal cortex. Similar evidence has been provided by a number of other studies; e.g., [12,13,14,15]. The reactivation of sensorimotor processes when the individual talks or thinks about an experience may take place not only in the visual system but also in other sensory systems and modalities; e.g., [4,5,16,17].

In the discussions of embodied language cognition, the embodiment of sentences that have a verb and describe an action has been the subject of a lot of debates and conflicting views. According to embodiment theories, when a literal sentence that includes a verb and describes an action is processed, that action is simulated; e.g., [1,4,5,6]. This means that the motor system is activated in the processing of that sentence. For example, in the processing of the literal sentence *he grasped the pen*, the action of grasping is simulated. This is done by the active role of the motor system that controls body movements. This is an example of embodied literal language cognition. In other words, the action is described by a literal sentence. However, the embodiment of metaphoric sentences that include a verb describing a metaphoric action, which sometimes is called a stronger version of embodiment or embodied metaphor processing, has been the subject of a larger volume of conflicting views [18]; for reviews, see [9,19]. According to this view, metaphor comprehension involves understanding one thing (the target of the metaphor) in terms of another thing (the base of the metaphor). Importantly, from this perspective, metaphorical understanding of one thing in terms of a body action (metaphoric action) involves a simulation of the metaphoric action [20]. Perhaps the most well-known example of such metaphors is the metaphorical phrase *grasp an idea*. In this metaphorical phrase, *understanding an idea* is metaphorically described in terms of *grasping a physical object*. It has been argued that in the processing of this metaphor, the base of the metaphor (understanding an idea) is understood as a physical action (grasping), and the physical action is simulated in the mind. This means that the motor system is actively employed to simulate this action during the processing of this metaphorical phrase. In other words, the main assumption of the embodied metaphoric language cognition is similar to the main assumption of embodied literal language cognition. That is, the same sensorimotor networks and sensorimotor processes involved in performing or perceiving the base of a metaphor (e.g., grasping an object) are also involved in processing the target of the metaphor and understanding the metaphor. Some studies have provided evidence that supports the main assumption of embodied metaphoric language cognition, including behavioral studies—e.g., [18,21,22,23]—and neuroimaging evidence—e.g., [24,25,26].

The simulation of literal and metaphoric actions may occur mentally, gesturally, or both at the same time. As mentioned, embodiment theories hold that when a literal sentence that refers to a literal action (e.g., push the door) is used, the action (e.g., pushing) is mentally simulated. This simulation may simultaneously occur mentally and gesturally (in co-speech gestures). This is also the case with metaphoric sentences that refer to a metaphoric action (e.g., grasp the idea). The mental simulation of the action of grasping may be accompanied by the gestural simulation of the metaphoric action of grasping. It has been argued that using co-speech gestures that depict literal and metaphoric actions show that people mentally simulate literal actions during the use of literal sentences and metaphoric actions during the use metaphoric sentences [27,28]. In McNeill’s typology [29] of gestures, a distinction is made between these two types of gestures. In this typology, these gestures are categorized as iconic gestures (literal gestures) and metaphoric gestures. A gesture that illustrates the shape of an object or a real action by the shape of the hand or the trace of hand movements is called an iconic gesture. An iconic gesture has a meaningful and non-arbitrary relationship with the shape of object or the action it refers to. For example, illustrating a triangle to refer to an object with a triangular shape or showing a pushing gesture to refer to the action of pushing a car are two examples of iconic gestures. A metaphoric gesture is a gesture that co-occurs with a metaphoric expression and illustrates the base of that metaphor. The base of the metaphoric expression may be an object, event, or action. A pushing gesture that co-occurs with the metaphoric expression *he pushed his argument* is an example of metaphoric gestures.

In this study, our aim was to examine how the embodiment of literal and metaphoric actions is affected when an adverb of manner is added to literal and metaphoric sentences. We specifically focused on the gestural simulation of literal and metaphoric actions when literal and metaphoric sentences included or did not include an adverb of manner. Before describing the methodology of our study, we briefly review a number of related works.

## 2. Studies on Gestural Simulations

One study [30] examined the impact of performing a gesture or imagining a gesture that illustrated the base of a metaphor on the understanding of that metaphor. Participants of this study first performed a metaphoric gesture or imagined a metaphoric gesture that illustrated the base of a metaphor. Immediately after performing or imagining the gesture, they read the metaphor and interpreted it. The results showed that participants performed better in interpreting metaphors after performing or even imagining a metaphoric gesture that illustrated the base of the metaphor. A recent related study [18] compared degrees of comprehension of three groups of participants in three different conditions: congruent gesture-prime conditions, opposite gesture-prime conditions, and no-prime conditions. In congruent gesture-prime conditions, the participants saw the gestural representation of a metaphor schema. Then, they read the metaphor and interpreted it. In opposite gesture-prime conditions, the participants saw a gesture that was opposite to the gestural representation of a metaphor schema. Then, they read the metaphor and interpreted it. The results showed that participants gave the best interpretation in congruent gesture-prime conditions.

In a recent study that was conducted on the basis of a suggested typology of metaphors (motion-based, static space-based, static object-based, and static event-based), the co-occurrence of metaphoric/beat gestures with each type of metaphors during the retelling of a set of stories was examined [31]. The results showed that among the four types of metaphors in the suggested typology, static space-based metaphors had the highest number of co-occurrence with metaphoric gestures. On the other hand, static event-based metaphors had the highest number of co-occurrence with beat gestures. Based on the results obtained in this study, it was suggested that spatial and motoric features of the base of a metaphor are the key factors that determine the type of a gesture used with a metaphor. Since motoric features are described by manner adverbs in some sentences, the use of adverbs with these metaphors could be one of the reasons behind the co-occurrence of these metaphors with a high number of gestures. In another study [32], the main idea of categorizing metaphors into four types in the previous study was also used to categorize literal sentences into four types. Then, the co-occurrence of metaphoric and iconic gestures with the four types of metaphoric sentences and the four types of literal sentences during retelling a set of stories was examined. The results showed that event-based metaphors had the lowest number of co-occurrence with metaphoric gestures, and event-based literal sentences had the lowest number of co-occurrence with iconic gestures. Such results again suggest spatial and motoric features play a key role in determining the type of gesture used with a literal or metaphoric statement.

The results of the reviewed studies confirm the idea of the mental simulation of actions described by literal and metaphoric sentences. Such simulation may be realized in co-speech iconic and metaphoric gestures. In this study, our aim was to examine how the embodiment of literal and metaphoric actions may be affected when a manner adverb is used in a literal or metaphoric sentence. In other words, we intended to find out how manner adverbs describing literal or metaphoric actions were embodied in co-speech gestures. Since the majority of past behavioral studies has focused on the embodiment of verbs, we specifically focused on adverbs to fill part of this gap in the literature of behavioral studies. We asked a group of Persian native speakers to read a set of stories and orally retell them in front of a camera. We examined the videos to find out how the use of manner adverbs that provided more information about literal or metaphoric actions might affect the use of gestures with literal and metaphoric sentences.

## 3. Method

### 3.1. Participants

We selected a group of students studying in Bartar Language Academy. The participants were selected randomly from a larger heterogeneous group of students who were available. This group consisted of 30 students, including 16 females and 14 males (age =16–25 years; Persian native speakers). All of them participated in the study voluntarily and gave their written informed consent.

### 3.2. Materials

Four Persian stories were used in this study. Each story had two versions. In one version of each story, four literal and four metaphoric sentences with verbs and without manner adverb were used. For example, the literal sentence *he was walking* and the metaphoric sentence *he pushed his ideas* were used in this version of the story. In the other version of the story, the same sentences were used with manner adverbs. For example, the literal sentence *he was walking fast* and the metaphoric sentence *he pushed his ideas forcefully* were used. The use of manner adverb with four literal sentences and four metaphoric sentences was the only difference between the two versions of each story. Therefore, four stories were without manner adverbs and four stories were with manner adverbs. Each story had 350–400 words. The written versions of these stories were used in this study. The English translations of literal and metaphoric sentences used in these stories have been given in Appendix A. The stories were told in Persian. The first story was about the hard life of a worker that made society aware of hardships of people living at lower levels of society. The second story was about a simple worker who later became the head of a large company. The third story was about the happy results of a sad event. The fourth story was about the value of honesty and trustworthiness. In addition, a camera was used to record participants’ gestures during oral retelling of stories.

### 3.3. Procedure

One day before conducting the main part of the study, the participants attended a training session. In this session, participants were given a sample written story and were asked to read it carefully and then retell it in front of a camera. For the main part of the study, participants were randomly divided into two groups of equal numbers (group A and group B). Just before conducting the main part of the study, the participants were provided with detailed oral instructions about the procedure of the study. However, to avoid any kind of impact on the performance of the participants during the retelling of the stories, purpose of the study was not revealed to the participants. The written version of story 1 without manner adverbs was given to the participants of group A. The written version of the same story with manner adverbs was given to the participants of group B. They were given twelve minutes to read it carefully. Then, each participant stood in front of a camera that had been installed two meters away. The participants had to retell the story in their own language and provide as many details as they could remember. They had five minutes to retell each story. The same procedure was used for story 2. However, for stories 3 and 4, the versions without manner adverbs were given to the participants of group B and the versions with manner adverbs were given to the participants of group A. Therefore, each participant in each group retold two stories without adverbs and two versions with adverbs.

### 3.4. Data Analysis

The videos were analyzed by researchers of the study. To avoid any bias in coding the data, two independent coders who were not involved in the study coded the gestures that were produced during the retelling of the stories. The inter-coder reliability was calculated by calculating Kappa coefficient to make sure that the process of coding gestures had an acceptable level of reliability. The numbers of literal/metaphoric sentences that had been produced without/with manner adverbs were obtained. Only gestures presenting a visual description of the action of the verbs were counted. In other words, iconic gestures depicting the action of literal sentences and metaphoric gestures depicting the action of metaphoric sentences were counted. Only those sentences produced during retelling the stories were counted, no matter what the original sentences were. For example, if a participant produced a metaphoric verb in a story that did not originally include a metaphoric verb, we counted it as one of the produced metaphoric verbs, because our aim was to examine the production of gestures during the production of such sentences. We used literal and metaphoric verbs (with/without adverbs) as stimuli to push participants to produce such sentences. However, we based our analysis on the sentences that were produced during the retelling of the stories. All sentences during the retelling of the stories were counted. Then, for each category, the number of cases where sentences had been used with iconic (literal sentences) or metaphoric (metaphoric sentences) gestures was obtained. In counting the gestures, only iconic and metaphoric gestures were counted. Moreover, the proportion of using gestures with sentences of each category was calculated. For example, for metaphoric sentences that had been produced with manner adverbs, the number of metaphoric gestures that accompanied the sentences was obtained. Then, the percentage of metaphoric sentences (without or with adverbs) that had been accompanied by gestures was calculated. The same method of analysis was used for other categories of sentences. A Chi-square test was used to examine the association between using manner adverbs in sentences and using gestures with the sentences. This analysis was done for both literal and metaphoric sentences separately. In addition, a t-test for independent samples was used to see whether the difference between using or not using adverbs implies significant difference in using or not using gestures.

## 4. Results

The results showed that in 95% of cases, the two coders were consistent in coding the data. In addition, the inter-coder consistency in the coding of the data was calculated by obtaining the Kappa coefficient. This coefficient was 0.90, which is a high level of reliability. The full results of the study have been presented in Table 1 and Table 2. Results presented in Table 1 show that in 53% of the cases, literal sentences that had been used with a manner adverb were accompanied by an iconic gesture. On the other hand, in 26% of the cases, literal sentences that had been used without adverbs were accompanied by an iconic gesture. The results of the Chi-square test showed a significant association between using manner adverbs in the literal sentences and using gestures (χ^2^ = 39.99, *p* <0.0001). This was confirmed by the results of a t-test for independent samples that compared the mean of literal sentences that included manner adverbs and were companied by iconic gestures and the mean of literal sentences that included manner adverbs and were not accompanied by iconic gestures (T-value = 3.91, df = 29, *p* < 0.001). These values show that when an adverb is used with a literal sentence, there is a higher possibility of using an iconic gesture than when the sentence is used without an adverb.

Interestingly, similar results were observed for metaphoric sentences. The results presented in Table 2 show that in 58.1% of the cases, metaphoric sentences that had been used with a manner adverb were accompanied by a metaphoric gesture. On the other hand, in 26% of the cases, metaphoric sentences that had been used without adverbs were accompanied by a metaphoric gesture. The results of the Chi-square test showed a significant association between using manner adverbs in the metaphoric sentences and using gestures (χ^2^ = 54.90, *p* <0.0001). This was confirmed by the results of a t-test for independent samples that compared the mean of metaphoric sentences that included manner adverbs and were companied by metaphoric gestures and the mean of metaphoric sentences that included manner adverbs and were not accompanied by metaphoric gestures (T-value = 4.08, df = 29, *p* < 0.001). These values show that when an adverb is used with a metaphoric sentence, there is a higher possibility of using a metaphoric gesture than when the sentence is used without an adverb.

These results show that using a manner adverb with a literal or metaphoric sentence increases the probability of using iconic/metaphoric gesture with the sentence. In the following section, we discuss these results from two rather different perspectives.

## 5. Discussion

The results of this study showed that when literal and metaphoric sentences include a manner adverb, there was a higher probability of using a gesture than when these sentences were used without manner adverbs. In other words, using a manner adverb increases the probability of using a gesture with literal and metaphorical sentences. A question that is raised here is why it happens. In this section, we discuss two explanations: one explanation is from a mainly cognitive perspective and another explanation is from a communicative perspective. The first explanation is based on the nature of the meanings of manner adverbs and the ways that they are embodied. As mentioned, from the perspective of embodiment theories, when a verb is used to describe a literal or metaphoric action, it is mentally simulated; e.g., [4,5,8]; for a review, see [9]. This mental simulation of literal and metaphoric actions may be realized in gestures [27,28]. An adverb used with a verb provides more information about the way that an action is done. Therefore, when a manner adverb is used with a verb, a more detailed simulation of the action may take place in the mind. For example, using the sentence *he was running* involves a mental simulation of the action of running; using the sentence *he was running fast* involves a mental simulation of the action of running with high speed. The latter simulation is more detailed and provides more information about the way that the action of running was performed. In this example, a more detailed simulation can mean that the process of simulation or embodiment of the action of running is stronger. In other words, a stronger degree of activity can take place in those neural networks that are involved in simulating the action of running. A more detailed simulation of the action and a stronger activity in the neural networks can explain why using manner adverbs increases the probability of using a gesture. This explanation is supported by some evidence from past studies. Some studies have provided evidence suggesting that using literal verbs (or thinking about literal actions) and even using metaphoric verbs (or thinking about metaphoric actions) leads to a mental simulation of literal/metaphoric actions; e.g., [3,6,18,25,30]. For example, using the sentence *he is walking* leads to a mental simulation of *walking* (literal action) and using the sentence *he grasped the idea* leads to a mental simulation of *grasping* (metaphoric action). This simulation initially takes place in the pre-motor areas. If this simulation is strong enough and passes a certain threshold, the simulation is spread to the primary motor areas and is realized in gestures [27,28]. Therefore, it can be suggested that when a verb is used without an adverb, the mental simulation of the verb is not very detailed and strong. It is restricted to the pre-motor areas and is not strong enough to spread to the motor areas. Therefore, the possibility of a gesture occurring is lower. When an adverb is used with the verb to provide detailed information about the action, the process of simulation may become more detailed and stronger. In such a situation, there would be a higher probability of the spreading of the activation to the primary motor areas.

Using a manner adverb with a verb means that a larger load of information about the quality of the verb is expressed and highlighted. In other words, the meaning of the verb and elements associated with it are put at the center of attention, and the semantic contents associated with the verb and manner adverb are embodied and simulated in the mind of the individual. The activation of a larger load of information associated with the verb can strengthen the embodiment of the verb. In other words, the degree of activity in the motor system and action simulation is strengthened. Since the activity in the neural networks associated with the embodiment of the action of the verb is in the interaction with the neural activity associated with the embodiment of the adverb, these two sets of related activities can strengthen each other.

The interesting point about the results of this study was that the higher use of gestures with manner adverbs took place for both literal and metaphoric sentences. This can be regarded as another evidence suggesting that the mechanisms of simulating literal and metaphoric actions are basically similar. Verbs of literal sentences (literal actions) are mentally simulated or embodied when literal sentences are used; similarly, verbs of metaphoric sentences (metaphoric actions) are simulated or embodied when metaphoric sentences are used. In the same way that literal verbs (literal actions) are simulated in the pre-motor areas and can spread to the primary motor areas by using a manner adverb, metaphoric verbs (metaphoric actions) are simulated in the pre-motor areas and can spread to the primary motor areas by using a manner adverb. In other words, although the literal and metaphoric meanings of a verb are very different, they are simulated and embodied through similar mechanisms, and their embodiments are affected in the same way when an adverb of manner is added to the sentences.

Since using a manner adverb adds some semantic content to the sentence, those neural networks that represent the semantic content of manner adverbs are activated. This means that using a manner adverb involves the activation of a larger part of the neural networks. For example, that part of the neural network that is activated by using the sentence *he was running fast* is larger than that part of the neural networks that is activated by using the sentence *he was running*. The first sentence activates a larger part because it contains more detailed meaning. In fact, some additional parts of the neural networks are activated to represent and embody the meaning of the added part. This proposal is supported by some neuroimaging works that have discussed the neural representations of the meaning of words and their embodiment in the neural networks; e.g., [2,4,5,7].

The second explanation for the higher use of gestures with manner adverbs in literal and metaphoric sentences is based on the quality of communication and the amount of information that is expressed. A manner adverb is used to provide more information about the verb and the action. Therefore, when a manner adverb is added to a literal or metaphoric sentence, a larger load of information is expressed. To convey this larger amount of information, the speaker may need to express it by more than one mode of communication. Gestures are perhaps the first available tool that can come into play to convey the additional load of information. Gestures are particularly useful in expressing spatial and motoric information as they can present a visual description of spatial and motoric features. Therefore, gestures are employed by speakers as effective tools to express information that is difficult to express in a verbal format. The simultaneous use of speech and gestures helps the speaker handle the process of expressing the intended meaning more effectively. In fact, gestures may be employed consciously or unconsciously by the speaker as a strategy to communicate more effectively.

## 6. Conclusions

The results of this study showed that adding a manner adverb to a sentence can strengthen the embodiment of the action described by the verb. This was shown by the higher use of gestures with literal and metaphoric sentences that included a manner adverb. This suggests that the strength of embodiment of some parts of a sentence may be affected by other parts of the sentence. A sentence may include a number of words referring to several different concepts. When the sentence is used, those concepts are embodied. The results of this study suggested that the embodiment of a given concept described by the sentence may be affected by the embodiment of other concepts in that sentence. In fact, it can be suggested that the embodiment of the whole of a situation that is described by a sentence is a dynamic process and a collection of the embodiment of concepts that interact in that sentence. Adding one element such as a manner adverb may significantly affect the embodiment of other parts of the sentence. In this study, adding a manner adverb strengthened the embodiment of the verb. This led to the spread of embodiment and simulation from the pre-motor areas (a fully mental simulation) to the primary motor areas and gestural simulation. This can happen not only for verbs and manner adverbs, but also for other parts of the sentence. In fact, the embodiment of a situation can be largely dependent on the words that are used to describe it. Certain types of words can strengthen the embodiment of the situation and push the speaker to use gestures.

Finally, like any other study, our study had some limitations in its scope. Not having access to people from a variety of languages was one of the most noticeable limitations of our study. Since there are some variations in the use of manner adverbs in different languages, there might be some variations in the use of gestures with adverbs in different languages. Conducting such a study on a larger group of people from a variety of linguistic backgrounds can produce more accurate and reliable results. Another limitation of our study was the context of collecting the data. We collected the data when participants were retelling a set of stories, which might not be a fully natural context. Collecting such a data in a more natural context can produce more valid and reliable results. In this study, we limited our investigation to the impact of using manner adverbs on the gestural embodiment of the verb (literal and metaphoric actions). The impact of other types of words on the embodiment of verbs is a question that can be examined in future research. Morever, our study examined this question on the basis of behavioral evidence. Looking at this question on the basis of neuroimaging evidence can be the subject of future neuroimaging studies.

## Figures and Tables

**Table 1:** Number and proportion of using iconic gestures with literal sentences (without or with manner adverb).

Produced Literal Sentences without Adverb	Produced Literal Sentences with Adverb	Produced Literal Sentences without Adverb Accompanied by Iconic Gesture	Produced Literal Sentences with Adverb Accompanied by Iconic Gesture
261	266	68 26%	141 53%

**Table 2:** Number and proportion of using metaphoric gestures with metaphoric sentences (without or with manner adverb).

Produced Metaphoric Sentences without Adverb	Produced Metaphoric Sentences with Adverb	Produced Metaphoric Sentences without Adverb Accompanied by Metaphoric Gesture	Produced Metaphoric Sentences with Adverb Accompanied by Metaphoric Gesture
212	203	47 22.1%	118 58.1%

## Data Availability

Data sharing is not applicable.

## Appendix A

**List of literal sentences without adverb**

1. He was walking
2. He climbed up
3. She entered the house
4. The car was moving
5. She shook her head
6. They began their campaign
7. She was doing her job
8. He approached the manager
9. They agreed
10. She was doing her difficult job
11. She was studying
12. He held the rope
13. He held the handle
14. They were looking at the conditions
15. They went into the factory
16. She came into the office

**List of literal sentences with adverb**

1. He was walking fast
2. He climbed up rapidly
3. She entered the house slowly
4. The car was moving fast
5. She shook her head slowly
6. They began their campaign vigorously
7. She was doing her job energetically
8. He slowly approached the manager
9. They agreed unanimously
10. She was doing her difficult job patiently
11. She was studying enthusiastically
12. He held the rope tightly
13. He held the handle tightly
14. They were looking at the conditions carefully
15. They went into the factory hurriedly
16. She proudly came into the office

**List of metaphoric sentences without adverb**

1. The life was moving
2. He pushed his ideas
3. He rose up in the company
4. She passed through the storm to reach her dream land
5. He faced with a mountain of obstacles
6. They were adhered to their beliefs
7. He was gaining the support
8. He fought against the problems
9. He rose up in the system
10. He was fighting against the obstacles
11. He was moving in a difficult road of challenges
12. His hands were tied by the management
13. She held her friends’ hands and did not leave them behind
14. Her strong self-confidence equipped her against the challenges ahead
15. Her attempts produced fruit
16. He was moving toward his goals

**List of metaphoric sentences with adverb**

1. The life was moving slowly
2. He pushed his ideas forcefully
3. He rose up rapidly in the company
4. She passed steadfastly through the storm to reach her dream land
5. He forcefully faced with a mountain of obstacles
6. They were firmly adhered to their beliefs
7. He was slowly gaining the support
8. He fought energetically against the problems
9. He gradually rose up in the system
10. He was patiently fighting against the obstacles
11. He was moving continuously in a difficult road of challenges
12. His hands were tied tightly by the management
13. She held her friends’ hands tightly and did not leave them behind
14. Her strong self-confidence heavily equipped her against the challenges ahead
15. Her attempts gradually produced fruit
16. He was firmly moving toward his goals
